# Effect of a Primary Care Walking Intervention with and without Nurse Support on Physical Activity Levels in 45- to 75-Year-Olds: The *P*edometer *A*nd *C*onsultation *E*valuation (PACE-UP) Cluster Randomised Clinical Trial

**DOI:** 10.1371/journal.pmed.1002210

**Published:** 2017-01-03

**Authors:** Tess Harris, Sally M. Kerry, Elizabeth S. Limb, Christina R. Victor, Steve Iliffe, Michael Ussher, Peter H. Whincup, Ulf Ekelund, Julia Fox-Rushby, Cheryl Furness, Nana Anokye, Judith Ibison, Steve DeWilde, Lee David, Emma Howard, Rebecca Dale, Jaime Smith, Derek G. Cook

**Affiliations:** 1 Population Health Research Institute, St George’s University of London, London, United Kingdom; 2 Pragmatic Clinical Trials Unit, Queen Mary’s University of London, London, United Kingdom; 3 Gerontology and Health Services Research Unit, Brunel University, London, United Kingdom; 4 Research Department of Primary Care & Population Health, University College, London, United Kingdom; 5 Department of Sport Medicine, Norwegian School of Sport Sciences, Oslo, Norway; 6 MRC Epidemiology Unit, University of Cambridge, Cambridge, United Kingdom; 7 Health Economics Research Group, Brunel, University of London, London, United Kingdom; 8 10 Minute CBT, Devonshire Business Centre, Letchworth Garden City, United Kingdom; The George Institute for Global Health, AUSTRALIA

## Abstract

**Background:**

Pedometers can increase walking and moderate-to-vigorous physical activity (MVPA) levels, but their effectiveness with or without support has not been rigorously evaluated. We assessed the effectiveness of a pedometer-based walking intervention in predominantly inactive adults, delivered by post or through primary care nurse-supported physical activity (PA) consultations.

**Methods and Findings:**

A parallel three-arm cluster randomised trial was randomised by household, with 12-mo follow-up, in seven London, United Kingdom, primary care practices. Eleven thousand fifteen randomly selected patients aged 45–75 y without PA contraindications were invited. Five hundred forty-eight self-reporting achieving PA guidelines were excluded. One thousand twenty-three people from 922 households were randomised between 2012–2013 to one of the following groups: usual care (*n* = 338); postal pedometer intervention (*n* = 339); and nurse-supported pedometer intervention (*n* = 346). Of these, 956 participants (93%) provided outcome data (usual care *n* = 323, postal *n* = 312, nurse-supported *n* = 321). Both intervention groups received pedometers, 12-wk walking programmes, and PA diaries. The nurse group was offered three PA consultations. Primary and main secondary outcomes were changes from baseline to 12 mo in average daily step-counts and time in MVPA (in ≥10-min bouts), respectively, measured objectively by accelerometry. Only statisticians were masked to group. Analysis was by intention-to-treat. Average baseline daily step-count was 7,479 (standard deviation [s.d.] 2,671), and average time in MVPA bouts was 94 (s.d. 102) min/wk. At 12 mo, mean steps/d, with s.d. in parentheses, were as follows: control 7,246 (2,671); postal 8,010 (2,922); and nurse support 8,131 (3,228). PA increased in both intervention groups compared with the control group; additional steps/d were 642 for postal (95% CI 329–955) and 677 for nurse support (95% CI 365–989); additional MVPA in bouts (min/wk) were 33 for postal (95% CI 17–49) and 35 for nurse support (95% CI 19–51). There were no significant differences between the two interventions at 12 mo. The 10% (1,023/10,467) recruitment rate was a study limitation.

**Conclusions:**

A primary care pedometer-based walking intervention in predominantly inactive 45- to 75-y-olds increased step-counts by about one-tenth and time in MVPA in bouts by about one-third. Nurse and postal delivery achieved similar 12-mo PA outcomes. A primary care pedometer intervention delivered by post or with minimal support could help address the public health physical inactivity challenge.

**Clinical Trial Registration:**

isrctn.com ISRCTN98538934.

## Abstract 2

In a cluster randomised clinical trial, Tess Harris and colleagues test the efficacy of an intervention intended to increase physical activity among predominantly inactive 45- to 75-year-olds in London.

## Author Summary

**Why Was This Study Done?:**

- Brisk walking for at least 30 min daily on five or more days weekly is a good way to achieve moderate-to-vigorous physical activity (MVPA) guidelines for health, yet many adults and older adults do not achieve these levels.
- Pedometers measure steps taken (step-count) and can increase walking and physical activity (PA) levels.
- Pedometer trials have usually measured short-term outcomes, combined pedometer effects with other support provided, and reported only step-counts, not time spent in MVPA.

**What Did the Researchers Do and Find?:**

- One thousand twenty-three inactive 45- to 75-y-olds from seven family (general) practices in London, UK, were randomly allocated to either a usual PA (control) group or to one of two intervention groups.
- The postal group were sent a pedometer, a PA diary, and instructions for a 12-wk walking programme to add in 3,000 steps or a 30-min walk on five or more days weekly; the nurse group received these materials through practice nurse PA consultations.
- Both intervention groups significantly increased their walking from baseline to 12 mo (step-counts increased by about 10% and time in MVPA increased by about one-third) compared to controls, with similar effect sizes for nurse and postal groups.

**What Do These Findings Mean?:**

- The findings suggest that a primary care pedometer intervention, delivered by post or with minimal support, could provide an effective way to increase PA levels in adults and older adults.

## Introduction

Physical activity (PA) helps adults remain healthy and improves physical function, quality of life, and emotional well-being [1]. Physical inactivity is the fourth leading risk factor for global mortality [2], leading to high health service costs [1, 3].

PA guidelines in adults and older adults advise at least 150 min of moderate-to-vigorous PA (MVPA) or 75 min of vigorous intensity PA weekly, or a combination of both, in at least 10-min bouts [1, 4, 5]. One way to achieve this is by 30 min of MVPA on at least 5 d weekly [1]. Although setting such goals is helpful, a graded dose–-response relationship exists for PA and health, so for inactive people any PA increase is valuable [6]. Emphasising that the MVPA can occur in 10- rather than 30-min bouts enables older adults and those with disabilities to increase their MVPA gradually. Walking is the most common adult PA; a pace of 5 km/hour qualifies as moderate intensity [7]. Walking is safe, as both frequency and intensity can be increased gradually [7]. Despite individual variation, moderate-intensity walking approximates 100 steps/min [8], or 3,000 steps in 30 min. Adding “3,000 steps in 30 min” onto habitual activity can increase step-counts [9] and reduce fasting glucose [9] in people with impaired glucose tolerance, but evidence for a change in MVPA in bouts is lacking. Reducing sedentary time may also be beneficial [1].

Programmes using personalised PA goals and behavioural strategies [10–12] can achieve PA increases. Cochrane Reviews called for PA interventions to include objective PA measurement [13, 14], adverse events [13], and comparisons of face-to-face with remote interventions [14]. Comparative evidence on individuals, couples, or households is also needed [15]. Systematic reviews of pedometer-based walking interventions showed increases of 2,000–2,500 steps/d [10, 16, 17]. However, studies were mainly small, volunteer based, and short term, the independence of pedometer effects were unclear, and outcomes focused on step-counts, not MVPA [10, 16, 17]. Primary care provides an ideal context for PA interventions, allowing population-based sampling, practice nurse involvement, and continuity of care. Brief PA advice in primary care is advocated [18]. However, to date, primary care has had little success in playing its part in the challenge of increasing population PA levels. Some small primary care pedometer-based walking interventions in older adults have increased PA levels at 3 [19], 6 [20], and 12 mo [21], but the effects of exercise referral schemes have been disappointing [22]. We therefore conducted a trial of a pedometer-based walking intervention in 45- to 75-y-olds, predominantly inactive, primary care patients, with novel separate evaluation of pedometer and nurse-support effects on objective PA outcomes, including MVPA in bouts. The research questions were as follows: (i) Does a 3-mo postal pedometer-based walking intervention increase PA in inactive 45- to 75-y-olds at 12 mo follow-up, and (ii) Do practice nurse PA consultations provide additional benefit? We also present effects on patient-reported outcomes, anthropometric measures, and adverse events. Cost-effectiveness analyses will be published separately.

## Methods

### Study Design and Participants

The trial protocol is published (S1 Text) [23]. A three-arm parallel cluster trial, randomised by household (allowing individuals and couples to participate) compared a 3-mo pedometer-based walking intervention, by post or with nurse support, with usual care. We recruited from an ethnically and socioeconomically diverse population in South London, UK, between September 2012 and October 2013, and follow-up was completed by October 2014. Six general (family) practices were selected, a seventh was later added, to ensure recruitment to target in the available time period. Eligible patients were 45–75 y old without contraindications to increasing MVPA. Care-home residents and those with unsuitable conditions were excluded [23]. All eligible participants were classified by household. Households were selected at random using Stata’s random number generator. All participants in single-person households were included. In multi-person households, an index person was selected at random, and a second person was randomly selected from amongst those aged within 15 y of the index person. Random samples of 400 eligible households were selected per practice [23], and individual invitations were posted. Those reporting achieving ≥150 min of MVPA weekly on a validated self-report PA question [24] were excluded. The London Research Ethics Committee (Hampstead) provided approval (12L/LO/0219). Trial registration: ISRCTN 98538934.

### Randomisation and Masking

Random allocation by household, avoiding couple contamination, was in a 1:1:1 ratio using the Kings College Clinical Trials Unit internet service, ensuring allocation concealment. Block randomisation was used within practice, with random-sized blocks for balanced groups and an even nurse workload. Participants, nurses, and researchers were unmasked to intervention allocation. Main outcome analyses were conducted by statisticians masked to study group.

### Procedures

Trial procedures, including individual informed written consent, baseline and 3- and 12-mo follow-up assessments, and complex intervention components are fully described elsewhere (S1 Text) [23] and summarised in S1 Fig. Of note, if participants were unable to be contacted at 3 mo, contact was still attempted again at the main 12-mo outcome. Assessment of outcomes were conducted identically for all three groups; an accelerometer (GT3X+, Actigraph LLC) was used for baseline, 3- and 12-mo masked PA assessment of step-counts, and time in different PA intensities. A simple pedometer, the SW-200 Yamax Digi-Walker, was used by both nurse and postal groups to record their own step-counts, as part of the intervention. The interventions incorporated behaviour change techniques (BCTs) and included individualised step-count, PA goals, and the “3,000-in-30” PA intensity message. Key intervention components were as follows: pedometers (SW-200 Yamax Digi-Walker); patient handbook; PA diary (including individual 12-wk walking plan); and three individually tailored practice nurse PA (10- to 20-min) consultations (nurse-support group only) were offered at approximately weeks 1, 5, and 9. The handbook and diary are available on the Pedometer and Consultation Evaluation (PACE-UP) website www.paceup.sgul.ac.uk/materials and both explain that adding 3,000 steps/d (approximating a 30-min walk) on five or more days weekly to an individual’s baseline step-count, progressing over 12 wk, would help achieve PA guidelines. BCTs, including goals and planning, self-monitoring and feedback, and encouraging social support, were included in the handbook, diary, and nurse consultations [23]. Participants in both postal and nurse intervention groups were encouraged to continue using the pedometer to monitor their walking and step-count beyond the 3-mo intervention period if they found this helpful. Control group participants were not provided with any feedback on their PA levels or materials promoting PA during the trial. They had follow-up assessments as per the intervention groups and were informed at the start of the trial that after 12-mo follow-up they would be offered feedback on their PA levels over the trial, a pedometer, a trial handbook, and a diary, either by post or as part of a single nurse consultation (according to their preference).

### Outcomes

The primary outcome is change in average daily step-count, assessed by accelerometry over 7 d, between baseline and 12 mo. Secondary PA outcomes (all accelerometry) are as follows: changes in step-counts between baseline and 3 mo; changes in time spent weekly in MVPA in ≥10-min bouts; and time spent sedentary between baseline and 3 and 12 mo.

Ancillary outcomes reported are as follows:

i) changes in anthropometry (body mass index, waist circumference, body fat) [23] at 12 mo;
ii) changes in patient-reported outcomes—exercise self-efficacy, anxiety, depression, health-related quality of life, pain (see protocol for full references [S1 Text] [23]) at 3 and 12 mo;
iii) adverse outcomes—falls, injuries, fractures, cardiovascular disease events, and deaths—assessed from trial monitoring procedures, questionnaires at 3 and 12 mo, and primary care records.

The following additional outcomes specified in the trial registry and trial protocol (S1 Text) will be published separately: economic (cost-effectiveness, including health service use outcomes and a Markov model to simulate long-term cost-effectiveness); self-report PA variables [23]; and a process evaluation. Qualitative evaluations from nonparticipants [25], participants [26], and practice nurses [27] are already published. An additional paper comparing trial participants and nonparticipants is also in progress.

### Statistical Analysis

A sample of 993 (331 per group) was required to detect a 1,000 steps/d difference (assuming a standard deviation of 2,700) at 12 mo when comparing any two groups, with 90% power, at *p* = 0.01. Household clustering was allowed for, assuming an intra-cluster correlation of 0.5 and an average household size of 1.6, and we assumed 15% attrition [23]. Analysis and reporting followed CONSORT guidelines (S2 Text).

Actigraph data were reduced using Actilife software (v 6.6.0), ignoring runs of ≥60 min of zero counts [23]. Vertical counts were used, as these are the basis of the validated step-count and MVPA algorithms. The analysis summary variables used were as follows: step-counts; accelerometer wear-time; time spent in MVPA (≥1,952 Counts Per Minute [CPM], equivalent to ≥3 Metabolic Equivalents [METs] [28]); time spent in ≥10-min MVPA bouts; and time spent sedentary (≤100 CPM, equivalent to ≤1.5 METs) [29].

Changes from protocol planned analyses (S1 Text) [23] were approved by the Trial Steering Committee prior to analyses. We report MVPA in ≥10-min bouts, as this relates more closely to PA guidelines [1, 4]. Only 20% of participants were nonwhite; ethnic group was therefore excluded from subgroup analyses due to low power.

To lessen attrition bias, our primary analysis included all participants with ≥1 d of 540 min wear-time at 12 mo. All analyses were carried out using Stata, version 12.0 [StataCorp]. Regression analyses used the *xtmixed* procedure. For accelerometry, this was in two stages. Stage 1 estimated average daily step-count at 12 mo and at baseline, derived by using the same two-level model (level 1 was day within individual, level 2 was individual) in which daily step-counts were regressed on day-order-of-wear and day-of-week. Random effects were assumed to be independent. In stage 2, we regressed estimated average daily step-count at 12 mo on estimated average daily baseline step-count, mo of baseline accelerometry, age, gender, general practice, and treatment group. This effectively measured change in step-count over the 12 mo, minimising bias and maintaining power. In this analysis, level 1 was individual and level 2 was household. The *pwcompare* (pairwise comparison) post estimation command was used to generate estimates and confidence limits for the difference in change between the nurse and control groups and the postal and control groups. The same command was used to provide a direct comparison of the nurse and postal groups; although the difference is effectively the difference of the previous two estimates, it is important to put confidence limits on this comparison. Secondary outcome measures, MVPA in ≥10-min bouts, and sedentary time were analysed using identical approaches, as were 3-mo outcomes. Checks confirmed that distributions of residuals from the regression models were normally distributed (S2 Fig). Change in anthropometric measures and patient-reported outcomes were estimated using identical models to stage 2 above. Sensitivity analyses were carried out for our primary outcome. We assessed (i) the effect of restricting analyses to those with ≥600 min of daily wear-time (both with ≥1 d of accelerometry at 12 mo and ≥5 d of accelerometry data at 12 mo); (ii) whether participants lost to follow-up, or who failed to record a single adequate day at 12 mo, might have introduced bias using the Stata procedure *mi impute*; (iii) the possible impact of outcomes not being missing at random; and (iv) the effect of adjusting for wear-time. We also conducted further analyses examining total time in MVPA, as opposed to time in MVPA in ≥10-min bouts.

### Patient Involvement

Pilot work with older primary care patients from three general practices was carried out ahead of seeking trial funding, with focus groups at each practice discussing ideas for a pedometer-based PA intervention. Patients were enthusiastic about the study and felt that the postal approach to recruitment and the interventions offered would be acceptable. They had input into aspects of the study design; for example, they encouraged us to offer the usual care arm a pedometer at the end of the follow-up period and they encouraged us to recruit couples as well as individuals, and to allow couples to attend nurse appointments together. A patient advisor was a Trial Steering Committee member and was involved in discussions about recruitment and study conduct, as well as advising about patient materials, dissemination of results to participants, and safety reporting mechanisms. All participants were provided with timely feedback of their individual trial results after completion of 12-mo follow-up, including their PA and body size measures over the trial duration. Summaries of results for the whole trial were disseminated to all trial participants as A4 feedback sheets after completion of baseline assessments and after analysis of the main results. A trial website (http://www.paceup.sgul.ac.uk/) has been created, and details have been circulated to participants. This also provides a summary of the trial results and details about further trial follow-up. All publications relating to the trial are provided on the website. The burden of the intervention was assessed by all participants in the nurse group with a questionnaire as part of the process evaluation and by samples of both intervention groups as part of the qualitative evaluation [26].

## Results

### Participants

**Fig 1:**
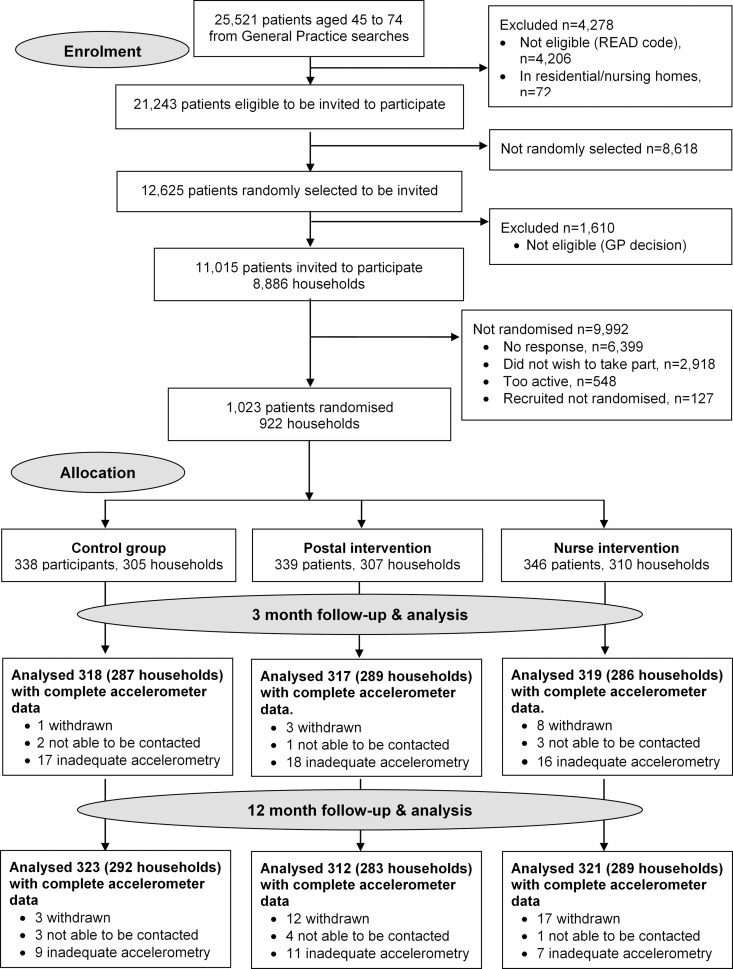
PACE-UP CONSORT diagram.

Of 11,015 invited, 6,399 did not respond, 548 were excluded due to self-reported PA guideline achievement, 127 were recruited but did not attend baseline assessment or provided inadequate baseline accelerometry data, and 1,023/10,467 (10%) were randomised (Fig 1). Of the 1,023 participants, 32 (3%) withdrew, and 8 (1%) were uncontactable at 12 mo. In total, 956/1,023 (93%) participants provided at least 1 d of 540 min wear-time accelerometer data and were included in 12-mo primary analyses. Baseline findings (Table 1) showed recruitment was balanced across age-groups; over a third were male. Characteristics were similar between groups. The nurse-support group had a slightly higher baseline adjusted average daily step-count (7,653, s.d. 2,826) and minutes spent weekly in MVPA in bouts of ≥10 min (105, s.d. 116) compared with the postal (steps 7,402, s.d. 2,476; MVPA in bouts 92, s.d. 90) and control groups (steps 7,379, s.d. 2,696; MVPA in bouts 84, s.d. 97). Overall, 218/1,023 (21%) achieved PA guidelines of ≥150 min of MVPA in bouts. Accelerometer wear-time was similar between groups at baseline and 3- and 12-mo follow-ups (Tables 1 and 2). Over 90% of all groups provided ≥5 d of ≥540 min wear-time at 12 mo (S1 Table).

**Table 1:** Baseline characteristics of 1,023 randomised participants.

	Control (*n* = 338)		Postal (*n* = 339)		Nurse (*n* = 346)	
	*n*	(%)	*n*	(%)	*n*	(%)
**Age at randomisation**						
45–54 y	101	(30%)	118	(35%)	121	(35%)
55–64 y	138	(41%)	125	(37%)	124	(36%)
65–75 y	99	(29%)	96	(28%)	101	(29%)
**Gender: Male**	115	(34%)	124	(37%)	128	(37%)
**Marital Status:** Married	213	(64%)	215	(65%)	230	(68%)
Randomised as a couple*	66	(20%)	68	(20%)	73	(21%)
**Employment status**						
In full or part-time employment	190	(57%)	193	(59%)	190	(56%)
Retired	102	(31%)	96	(29%)	101	(30%)
Other	39	(12%)	39	(12%)	50	(15%)
**NS-SEC (current or previous job)**						
Higher managerial, administrative, professional	199	(62%)	191	(60%)	184	(56%)
Intermediate occupations	70	(22%)	85	(27%)	95	(29%)
Routine and manual occupations	51	(16%)	44	(14%)	52	(16%)
**Ethnicity**						
White	253	(78%)	270	(83%)	267	(80%)
Black/African/Caribbean/Black British	30	(9%)	31	(10%)	40	(12%)
Asian/Asian British	26	(8%)	20	(6%)	22	(7%)
Other	15	(5%)	4	(1%)	6	(2%)
**Current smoker**	27	(8%)	29	(9%)	26	(8%)
**General Health**^**†**^: Very Good or Good	265	(80%)	277	(84%)	277	(82%)
**Chronic diseases**^**†**^						
None	129	(39%)	135	(41%)	117	(35%)
1–2	183	(55%)	171	(51%)	188	(55%)
≥3	21	(6%)	27	(8%)	34	(10%)
**Presence of self-reported pain**^**†**^	220	(66%)	236	(71%)	234	(70%)
**Limiting long-standing illness**^**†**^	76	(23%)	73	(22%)	74	(22%)
**Townsend disability score**^**†**^						
None (0)	190	(57%)	196	(59%)	210	(62%)
Slight or some disability (1–6)	127	(38%)	130	(39%)	124	(36%)
Appreciable or severe disability (7–18)	15	(5%)	8	(2%)	7	(2%)
**HADS depression score**^**†**^: borderline or high	36	(11%)	33	(10%)	42	(12%)
**HADS anxiety score**^**†**^: borderline or high	65	(19%)	64	(19%)	71	(21%)
**Low self-efficacy score**^**†**^	102	(31%)	96	(29%)	117	(35%)
**Month of baseline measure**						
March–May	80	(24%)	75	(22%)	76	(22%)
June–August	105	(31%)	106	(31%)	110	(32%)
September–November	88	(26%)	82	(24%)	92	(27%)
December–February	65	(19%)	76	(22%)	68	(20%)
**Physical characteristics**						
Overweight/obese: BMI ≥25kg/m^2^	227	(67%)	221	(65%)	233	(67%)
	**Mean**	**(s.d.)**	**Mean**	**(s.d.)**	**Mean**	**(s.d.)**
Fat mass (kg)	26	(10)	27	(11)	26	(11)
Waist circumference (cm)	93	(14)	94	(14)	93	(13)
**Accelerometry data**	**Mean**	**(s.d.)**	**Mean**	**(s.d.)**	**Mean**	**(s.d.)**
Adjusted baseline step-count per day	7,379	(2,696)	7,402	(2,476)	7,653	(2,826)
Total weekly minutes of moderate or vigorous physical activity (MVPA) in ≥10-min bouts	84	(97)	92	(90)	105	(116)
Average daily sedentary time (minutes)	613	(68)	614	(71)	619	(78)
Average daily wear time (minutes)	789	(73)	787	(78)	797	(84)

*two participants in the postal group and one in the nurse groups were randomised and took part in the trial as a couple, although their partners were excluded before randomisation due to lack of wear-time.

**†**Full references for General Health, Chronic disease score, self-reported pain, HADS depression and anxiety scores, Townsend Disability Score, and Self-Efficacy Score are given in the trial protocol [23].

Abbreviations: BMI, body mass index, HADS, hospital anxiety and depression scale; NS-SEC, National Statistics Socioeconomic Classification.

**Table 2:** Primary and secondary accelerometry outcome data.

	Postal versus Control			Nurse versus Control			Nurse versus Postal		
	Effect	95% CI	*p*-value	Effect	95% CI	*p*-value	Effect	95% CI	*p*-value
**Daily step count**									
**3 mo**	692	(363, 1,020)	<0.001	1,172	(844, 1,501)	<0.001	481	(153, 809)	0.004
**12 mo**	642	(329, 955)	<0.001	677	(365, 989)	<0.001	36	(-277, 349)	0.82
**Total weekly minutes of MVPA in ≥10-min bouts**									
**3 mo**	43	(26, 60)	<0.001	61	(44, 78)	<0.001	18	(1, 35)	0.04
**12 mo**	33	(17, 49)	<0.001	35	(19, 51)	<0.001	2	(-14, 17)	0.83
**Daily sedentary time (minutes)**									
**3 mo**	-2	(-12, 7)	0.59	-7	(-16, 3)	0.16	-4	(-13, 5)	0.38
**12 mo**	1	(-8, 10)	0.83	-0.2	(-9, 9)	0.96	-1	(-10, 8)	0.79
**Daily wear time (minutes)**									
**3 mo**	2	(-8, 12)	0.69	4	(-6, 14)	0.40	2	(-8, 12)	0.65
**12 mo**	9	(-1, 19)	0.08	9	(-0.8, 19)	0.07	0.3	(-10, 10)	0.96

Results shown for “Postal versus Control” are the additional effect seen in the postal group relative to the control group and similarly for “Nurse versus Control” and “Nurse versus Postal.”

Accelerometry data were available in the control, postal, and nurse groups, respectively, for 318, 317, and 319 participants at 3 mo and for 323, 312, and 321 at 12 mo.

All models include practice, gender, age at randomisation, and month of baseline accelerometry as fixed effects and household as a random effect in a multi-level model.

The *xtmixed* command in Stata v12 was used, followed by the post-estimation command *pwcompare* to generate the pairwise estimates of effects and their confidence intervals.

Among intervention participants, 256/346 (74%) of the nurse-support group attended all three sessions and 268/339 (79%) of the postal and 281/346 (81%) of the nurse-support group sent back PA diaries completed with their pedometer step-counts after the intervention.

### Effect of the Intervention on PA at 3 and 12 Mo

#### Three-mo (interim) outcomes (Table 2)

There were significant differences for change in step-counts from baseline to 3 mo between intervention groups and the control group: additional step-counts (steps/day) postal 692 (95% CI 363, 1,020; *p* < 0.001), nurse-support 1,172 (95% CI 844, 1,501; *p* < 0.001); the difference between the intervention groups was statistically significant: 481 (95% CI 153, 809; *p* = 0.004). Findings for MVPA showed a similar pattern: additional MVPA in bouts (min/wk) postal 43 (95% CI 26, 60; *p* < 0.001), nurse-support 61 (95% CI 44, 78; *p* < 0.001); the difference between intervention groups was 18 (95% CI 1, 35; *p* = 0.04). Sedentary time was similar between groups. Summary data for 3-mo PA outcomes are shown in S2 Table.

#### Twelve-mo (main) outcomes (Table 2)

Both intervention groups increased their step-counts at 12 mo compared with controls: additional step-counts (steps/day) postal 642 (95% CI 329, 955; *p* < 0.001) and nurse-support 677 (95% CI 365, 989; *p* < 0.001), with no statistically significant difference between intervention groups, 36 (-277, 349). Time spent in MVPA in bouts showed a similar pattern; both intervention groups increased at 12 mo compared with controls; additional MVPA in bouts (min/wk) postal 33 (95% CI 17, 49; *p* < 0.001) and nurse-support 35 (95% CI 19, 51; *p* < 0.001), with no statistically significant difference between intervention groups 2 (-14, 17). Sedentary time was similar between groups. Summary data for 12-mo PA outcomes are shown in S2 Table.

### Effect of the Intervention on Other Health-Related Outcomes

Fat mass was slightly reduced at 12 mo in both intervention groups, but these differences did not differ significantly from the control group (Table 3). There was no change in body mass index or waist circumference. The interventions had no significant effects on anxiety, depression, health-related quality of life, or pain scores at 3 or 12 mo. Exercise self-efficacy score significantly increased in both intervention groups at 3 mo compared with controls, and there was a greater effect in the nurse group compared with postal. By 12 mo, there was a difference in self-efficacy score between only the nurse and control groups; the postal group was intermediate between, but not significantly different from, the other groups (Table 3). Summary data for health-related outcomes are shown S2 Table.

**Table 3:** Ancillary outcomes.

	Postal versus Control			Nurse versus Control			Nurse vs Postal		
	Effect	95% CI	*p*-value	Effect	95% CI	*p*-value	Effect	95% CI	*p*-value
**BMI (kg/m**^**2**^**)**									
**12 mo**	-0.1	(-0.3, 0.1)	0.24	-0.03	(-0.2, 0.1)	0.71	0.07	(-0.1, 0.3)	0.42
**Fat Mass (kg)**									
**12 mo**	-0.4	(-0.8, 0.07)	0.10	-0.2	(-0.7, 0.2)	0.30	0.1	(-0.3, 0.6)	0.54
**Waist circumference (cm)**									
**12 mo**	-0.04	(-0.8, 0.7)	0.92	0.08	(-0.6, 0.8)	0.23	0.1	(-0.6, 0.8)	0.74
**HADS Anxiety Score**									
**3 mo**	-0.3	(-0.7, 0.1)	0.13	-0.3	(-0.7, 0.1)	0.16	0.01	(-0.4, 0.4)	0.94
**12 mo**	-0.2	(-0.6, 0.2)	0.28	-0.2	(-0.6, 0.2)	0.28	0.0006	(-0.4, 0.4)	1.00
**HADS Depression Score**									
**3 mo**	-0.2	(-0.6, 0.1)	0.12	-0.2	(-0.5, 0.1)	0.19	0.04	(-0.3, 0.3)	0.82
**12 mo**	-0.1	(-0.5, 0.2)	0.44	-0.02	(-0.4, 0.3)	0.91	0.1	(-0.2, 0.5)	0.51
**EQ5D**									
**3 mo**	-0.005	(-0.02, 0.01)	0.60	-0.01	(-0.03, 0.01)	0.26	-0.006	(-0.03, 0.01)	0.54
**12 mo**	-0.01	(-0.03, 0.01)	0.30	-0.01	(-0.03, 0.01)	0.23	-0.002	(-0.02, 0.02)	0.87
**Exercise self-efficacy**									
**3 mo**	1.1	(0.2, 2.0)	0.01	2.3	(1.4, 3.2)	<0.001	1.2	(0.3, 2.1)	0.01
**12 mo**	0.6	(-0.3, 1.6)	0.20	1.2	(0.3, 2.2)	0.01	0.6	(-0.4, 1.5)	0.22
**Self-report pain**									
**3 mo**	0.05	(-0.06, 0.17)	0.37	0.05	(-0.07, 0.16)	0.42	-0.004	(-0.12, 0.11)	0.94
**12 mo**	0.05	(-0.06, 0.17)	0.35	0.02	(-0.10, 0.13)	0.76	-0.04	(-0.15, 0.08)	0.53

Results shown for “Postal versus Control” are the additional effects seen in the postal group relative to the control group and similarly for “Nurse versus Control” and “Nurse versus Postal.”

At baseline, data were available for all participants for BMI and waist circumference, and for 335, 337, and 346 participants in the control, postal, and nurse groups, respectively, for fat mass.

At 12 mo, data were available in the control, postal, and nurse groups, respectively, for 323, 314, and 321 participants for BMI and waist circumference, and for 319, 308, and 320 for fat mass.

Questionnaire data were available for varying numbers of participants at baseline, 3 mo, and 12 mo.

All models include practice, gender, age at randomisation, and month of baseline accelerometry as fixed effects and household as a random effect in a multi-level model.

Full references for HADS depression and anxiety scores, EQ5D, Exercise Self-Efficacy Score, and self-reported pain are given in the trial protocol [23].

Abbreviations: BMI, body mass index; EQ5D, health-related quality of life; HADS, hospital anxiety and depression scale.

#### Subgroup analyses

**Fig 2:**
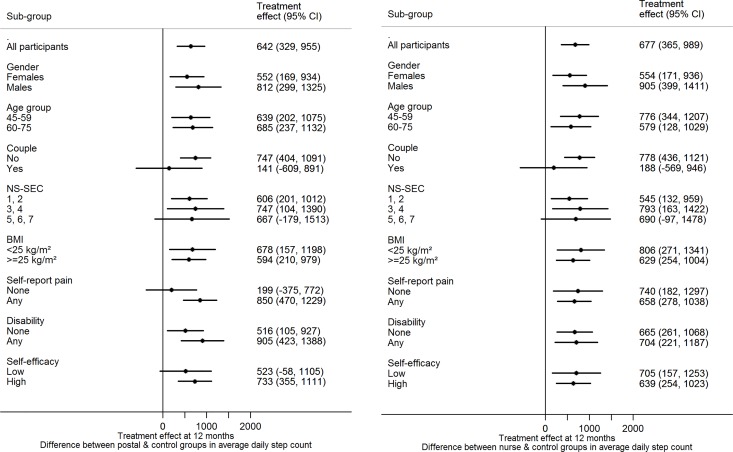
Treatment effect for primary outcome by subgroup at 12 mo. (a) Postal and control groups (b) nurse and control groups. Abbreviations: BMI, body mass index; NS-SEC, National Statistics Socioeconomic Classification.

There was no evidence of effect modification on change in step-count at 12 mo for either of the intervention groups versus control for any of the following factors: age, gender, taking part as a couple, body mass index, disability, pain, socioeconomic group, exercise self-efficacy (Fig 2).

### Effect of the Intervention on Adverse Events and Serious Adverse Events

Total adverse events did not differ between groups at 3 or 12 mo whether self-reported on the questionnaire (falls, fractures, sprains, and injuries) or from primary care records (any adverse event) (Table 4). There was also no between-group difference in trial serious adverse events reported for safety monitoring. Self-reported falls were lower in the nurse group at 12 mo (*p* = 0.02). Falls reported in primary care records over 12 mo are fewer, but also in the same direction, although differences are nonsignificant (*p* = 0.13). Primary care recorded cardiovascular events over 0–12 mo were lower in the intervention groups than in controls (*p* = 0.04).

**Table 4:** Adverse events.

	0–3 mo								0–12 mo							
	*n*	Control	(%)	Postal	(%)	Nurse	(%)	*p*-value^†^	*n*	Control	(%)	Postal	(%)	Nurse	(%)	*p*-value^†^
**Adverse events reported on the questionnaire**																
Fall, fracture, sprain, or injury	931	59/313	(19)	70/310	(23)	65/308	(21)	0.51	946	113/318	(36)	99/310	(32)	96/318	(30)	0.34
Fall		25	(8)	24	(8)	24	(8)	0.99		71	(22)	57	(18)	43	(14)	0.02
Fracture		3	(1)	3	(1)	7	(2)	0.28		15	(5)	10	(3)	11	(3)	0.57
Sprain or injury		49	(16)	54	(17)	47	(15)	0.74		66	(21)	68	(22)	63	(20)	0.81
Deterioration in health problems already present since start of study	911	33/311	(11)	30/303	(10)	39/297	(13)	0.42	924	68/313	(22)	67/300	(22)	65/311	(21)	0.91
**Adverse events from primary care records**^**‡**^																
Any adverse event	1005	29/334	(8.7)	23/331	(7.0)	20/340	(5.9)	0.36	1005	85/334	(25.5)	75/331	(22.7)	77/340	(22.7)	0.62
Cardiovascular^§^		2	(0.6)	0		1	(0.3)	0.55		8	(2.4)	1	(0.3)	2	(0.6)	0.04
Fracture		4	(1.2)	2	(0.6)	2	(0.6)	0.68		11	(3.3)	4	(1.2)	4	(1.2)	0.11
Sprain/injury		2	(0.6)	1	(0.3)	2	(0.6)	1.00		8	(2.4)	4	(1.2)	5	(1.5)	0.51
Fall		0		0		0				8	(2.4)	4	(1.2)	2	(0.6)	0.13
Pain (back or lower limb)		23	(6.9)	20	(6.0)	16	(4.7)	0.48		65	(19.5)	65	(19.6)	70	(20.6)	0.93
Serious adverse event spontaneously reported*	1,023	3/338	(0.9)	1/339	(0.3)	3/346	(0.9)	0.65	1023	10/338	(3.0)	5/339	(1.5)	11/346	(3.2)	0.30

† Chi-squared tests or Fisher exact tests were carried out to assess statistical significance for overall differences between the three groups.

‡ 1,005 participants gave permission at randomisation for their primary care records to be accessed and downloaded.

§ Cardiovascular events recorded in primary care records included a new episode of any of the following: myocardial infarction, coronary artery bypass graft, angioplasty, ischaemic heart disease, angina, transient ischaemic attack,and stroke.

* Information on spontaneously reported serious adverse events were collected for the entire cohort, *n* = 1,023. Serious adverse events were recorded for safety purposes contemporaneously in the trial and included the following: deaths, hospital admission, and new onset disability. All the serious adverse events reported during the 0–12 mo trial follow-up were emergency hospital admissions.

#### Sensitivity analyses and imputations

Restricting analyses to those with ≥600 min daily wear-time (and either ≥1 or ≥5 d of accelerometry data at 12 mo) and imputations with both missing at random and missing not at random assumptions and analyses, adjusting for accelerometer wear-time, gave broadly similar effect size estimates for both interventions compared with control and to each other and made no difference to interpretation (S3 Table).

Analyses of total MVPA as the outcome produced almost identical effect size estimates as found with MVPA in ≥10-min bouts; at 12 mo, postal versus control was 36 (95% CI 17, 55) min/wk and nurse versus control was 32 (95% CI 13, 50) min/wk. In other words, all of the increase in MVPA was in ≥10-min bouts.

## Discussion

### Principal Findings

The interventions increased objectively assessed PA (step-counts by about 650–700 steps per day and MVPA in bouts by about 33–35 min/wk) among predominantly inactive 45- to 75-y-olds at 12 mo. Whilst nurse delivery had a greater effect than postal delivery at 3 mo, by 12 mo this difference was not sustained. Exercise self-efficacy was significantly increased by both interventions compared to control at 3 mo and in the nurse group at 12 mo. The interventions had no effect on sedentary time, anthropometry, or other outcomes and did not increase adverse events. Both interventions were well accepted; three-quarters of the nurse group attended all three sessions and ~80% of both groups returned completed step-count diaries. The trial was novel in clearly separating out the effects of pedometer provision and nurse support in a general population sample of adults and older adults and demonstrating the effects on both step-counts and MVPA in bouts, thus making the outcome assessment relevant to current national and international PA guidelines.

### Study Strengths and Limitations

Study strengths include the following: a large, population-based, primary care sample; household randomisation, allowing comparison of individual and couple effects; three arms, allowing separation of nurse support and pedometer/handbook effects; practice nurses, rather than researchers or exercise specialists, delivering the intervention; good uptake of nurse appointments and return of completed step-count diaries; an objective PA outcome, relevant to PA guidelines; adverse event measurement from primary care records; a 93% follow-up rate; and embedded economic and qualitative evaluations (not presented here). There were some study limitations. The 10% (1,023/10,467) recruitment rate raises issues of generalizability, which are dealt with in the later section on Implications for Policy, Practice, and Future Research. At baseline, 218/1,023 (21%) achieved PA guidelines based on accelerometry. They were not excluded because, if rolled out in primary care, self-report would define participation. Our nurse intervention group had slightly higher baseline PA levels; however, results were not biased, as analyses were based on individual change, controlling for baseline PA level. It was impossible to mask participants and nurses to group and, pragmatically, research assistants recruited and followed up the same participants, so they were unmasked to group at outcome assessment. However, all the primary and secondary PA outcomes were assessed objectively. Participants might have tried harder with their PA when monitored, but this would also have affected controls and would be reduced by using a 7-d protocol [16]. Also, our intervention groups increased MVPA in bouts of ≥10 min, implying that participants made changes suggested by the programme. Despite recruiting to target and having excellent follow-up, our confidence intervals for the difference between intervention groups cannot rule out a small 12-mo difference.

### Main Results in Context of Other Literature

To our knowledge, this is the largest population-based trial of a pedometer-based walking intervention with 12-mo follow-up and is consistent with our findings in 60- to 75-y-olds in the smaller PACE-Lift trial [21]. Whilst the PACE-Lift intervention also included pedometer feedback, step-count diary, and practice nurse PA consultations based around BCTs, it comprised four longer consultations, which also included accelerometer feedback on PA intensity. PACE-Lift only had a single intervention arm and was therefore unable to separate out PA monitor effects from those of the nurse support. Despite a less intense intervention, PACE-UP has delivered similar levels of effect at both 3 and 12 mo and additionally has shown what can be achieved via a postal route. Compared with systematic reviews [10, 16, 17], our absolute step-count increase was modest. However, most trials with 12 mo of data have been based on small numbers and either volunteers [30], high-risk groups [9], or self-report PA data [31], likely leading to larger effects. PA guidelines focus on time in MVPA, not step-counts; the reviews presented no data on this important outcome [10, 16, 17]. PACE-UP results confirm PACE-Lift findings [21], with significant 12-mo increases in MVPA in bouts. Based on the “3,000-in-30” formula, 35 extra min of MVPA/wk in bouts corresponds to 500 extra steps/day. Thus, three-quarters of the extra steps achieved contributed to MVPA in bouts. We believe our trial is the first to show that the “3,000-in-30” message [8] can lead to an approximately one-third increase in weekly MVPA in bouts at 12 mo, achieved across both intervention groups. It is also reassuring that our interventions did not increase sedentary time, given its potential harm, as compensation can sometimes occur.

Most pedometer-based interventions have not separated pedometer and support effects [14, 16, 21]. The Healthy Steps trial showed pedometers achieved an additional effect compared with a primary care PA prescription, but PA outcomes were self-reported [31]. PACE-UP demonstrates that whilst the nurse intervention group had a significantly greater effect on both step-counts and time in MVPA at 3 mo, by 12 mo both nurse and postal interventions still had a significant effect, but with no evidence of difference between them. This stronger effect during the period of contact with the nurse, which was not sustainable longer term, has also been shown in other interventions with health professionals [32]. Both nurse and postal groups received a pedometer, diary, and handbook as part of the PACE-UP package; it is not possible to know how much the individual components contributed. A systematic review suggested that step-count diaries were common to successful pedometer interventions [16], and approximately 80% of both of our intervention groups returned completed step-count diaries. Also, our qualitative findings suggest that participants from both groups valued the handbook and diary as well as the pedometer [26].

We found no effect of the interventions on body mass index or fat mass, consistent with other studies [21, 30]. Our interventions did not affect anxiety or depression scores, consistent with other primary care pedometer-based interventions, suggesting either no effect or insensitivity of these measures to change, particularly when levels are in the normal range for most people [19, 21]. However, whilst a few participants mentioned negative effects from overdoing walking, most intervention participants talked about feeling fitter, sleeping better, improved mood, having more energy and less pain, and keeping more active into older age [26]. There is a lack of data comparing individual, couple, or household participation in walking studies [15, 21]. Household sampling allowed us to investigate this, but only 20% participated as couples, reducing the power of our subgroup analyses, which showed no effect.

Self-efficacy differences between both intervention groups and controls at 3 mo and between the nurse group and controls at 12 mo are consistent with the positive relationship between changing self-efficacy and PA behaviour [33]. The BCTs most associated with self-efficacy and successful outcomes are goal and action planning, prompting self-monitoring and feedback, and planning of social support/change [33]. All these BCTs were specifically recommended in recent guidance [11] and were included in our study in written materials for both intervention groups and as a focus of nurse PA consultations [23]. Our qualitative interviews found that more BCT comments were made by the nurse than postal group, apart from around self-monitoring [26]. Increased self-efficacy is important for long-term PA adherence [34].

Walking is a safe intervention indicated in many chronic diseases [1, 7], although empirical data are limited [13] and a large trial on 40- to 74-y-old women encouraging a single 30-min brisk walk 5 d weekly reported increased falls and injuries [24]. Our findings showing no increase in adverse events builds on similar evidence from PACE-Lift [21], using both self-report and primary care data, and highlights the potential importance of building up MVPA gradually, particularly in those who are inactive or have comorbidities [1, 6]. The suggestion of a protective effect of the interventions on falls and cardiovascular events is plausible, but not definitive, as it is based on small numbers of events.

### Implications for Policy, Practice, and Future Research

Individual PA behaviour change approaches such as PACE-UP are important in tackling the public health challenge of physical inactivity but for maximum benefit need to occur alongside environmental and policy approaches [12]. Our results support current guidance for pedometers, which suggests that they are used as part of a package that includes support to set realistic goals, monitoring, and feedback [35]. Only 10% of eligible individuals were randomised, similar to other primary care PA trials [19, 36] but lower than the 30% in our recent older adult trial [21]. However, 10% of a population sample is still a very useful percentage to be participating in a public health intervention, and this trial shows the potential of primary care to contribute to PA public health goals. It is important to consider whether the participants randomised are representative of the target population from which they were drawn, particularly given the uptake rate of 10%. From Table 1, we can see that, of those randomised, there were more women than men, and the proportion of participants of Asian origin and from deprived areas was low and fewer than expected from the areas sampled. While approximately 4/5 of those randomised reported their health as good or very good, about 2/3 were overweight or obese, half reported one to two chronic diseases, nearly 2/5 reported slight/some disability, and over 1/5 reported a limiting, longstanding illness. Older adults were well represented. Thus, although it is unlikely that those randomised are entirely typical of the practice populations (it would be surprising if they were), there was substantial representation from groups who are particularly likely to benefit from the intervention, specifically older adults, women, and the overweight. Moreover, 1/3 of those randomised rated their self-efficacy for exercise as low. Nevertheless, some groups, for example Asians, will be underrepresented, and we are carrying out further work comparing participants and nonparticipants to identify these. Tailoring future interventions to be more acceptable to such groups will be important. If the intervention were to be rolled out in routine primary care, take-up could be higher, with no requirement for informed consent, randomisation, and rigorous evaluation. Handing out the intervention materials (pedometer, handbook, and diary) in primary care consultations where advice to increase low PA levels is already being offered is also likely to increase the intervention’s reach (e.g., in relevant chronic disease consultations or as part of preventive health checks, such as the UK National Health Service Health Checks, which cover a similar age-group and aim to reduce cardiovascular risk [37]). The intervention could also be a valuable addition to diabetes prevention strategies, such as the National Health Service Diabetes Prevention Programme [38], where primary care is being used to identify patients at high risk of developing diabetes, the majority of whom are inactive. The “3,000 steps-in-30 min” neatly captures intensity and could become a commendable new public health goal, with many people now having the ability to measure steps easily with their mobile phones.

Our interventions led to an extra 33–35 min weekly of MVPA in bouts (an increase of about a third from baseline) and an extra 642–692 steps per day in a predominantly inactive cohort. Based on a systematic review, which has quantified the strength of association between PA (particularly walking) and developing coronary heart disease [39], the increase of 33 min/wk in the postal group in our study at 12 mo, if sustained, would be expected to reduce coronary heart disease risk by 4.5% (95% CI 3%, 6%; see S3 Text for details). Similarly, a cohort study relating pedometer-measured steps to mortality [40] allowed us to estimate that a sustained increase of 642 steps/day would be expected to decrease all-cause mortality by 4% (95% CI 1%, 7%).

Whilst the nurse intervention produced greater effects at 3 mo, by 12 mo both interventions performed similarly. However, maintenance is important to consider, as long-term health effects require sustained PA increases and little is known about the effectiveness of PA interventions beyond 12 mo [13, 16]. We designed both PACE-UP interventions to have lasting effects [23], including techniques shown to help maintain behaviour change (e.g., encouraging feedback and self-monitoring; relapse prevention strategies and “if-then” plans in case of relapse; building social support; and incorporating new behaviours into daily routines [11]). Some strategies may have been more effective in the nurse group; the sustained self-efficacy difference between nurse and control groups at 12 mo supports this possibility. It is therefore important to test the long-term effectiveness of both interventions, and we are currently following up the PACE-UP cohort at 3 y.

## Conclusion

The PACE-UP pedometer-based walking intervention increased step-counts by approximately a tenth and time in MVPA in bouts by a third in predominantly inactive 45- to 75-y-old primary care patients. Nurse delivery over three consultations had no greater effect on 12-mo PA outcomes than postal delivery. A primary care pedometer intervention, delivered by post or with minimal contact, would provide an effective approach to addressing the public health physical inactivity challenge.

## Supporting Information

S1 Text PACE-UP trial protocol. (PDF)

S2 Text PACE-UP CONSORT checklist. (DOCX)

S3 Text Details on cardiovascular and overall mortality risk reduction. (DOCX)

S1 Fig Trial procedures and complex intervention components. (TIF)

S2 Fig Residuals from 12-mo models for steps and weekly MVPA in ≥10-min bouts. (TIF)

S1 Table Number of days with ≥540 min accelerometer wear-time by treatment group at baseline, 3 mo, and 12 mo. (DOCX)

S2 Table Summary data for main outcome and ancillary outcome variables. (DOCX)

S3 Table Sensitivity and imputation analyses for the primary outcome (step-count at 12 mo). (DOCX)

## Abbreviations

BCTs: behaviour change techniques
CPM: counts per minute
MET: metabolic equivalent
MVPA: moderate-to-vigorous physical activity
PA: physical activity
PACE-UP: Pedometer and Consultation Evaluation
s.d: standard deviation.
