# Supplementary material for: Effect of a Primary Care Walking Intervention with and without Nurse Support on Physical Activity Levels in 45- to 75-Year-Olds: The Pedometer And Consultation Evaluation (PACE-UP) Cluster Randomised Clinical Trial
Source: PLoS Med. 2017 Jan 3;14(1):e1002210. doi: 10.1371/journal.pmed.1002210 (PMC5207642; doi:10.1371/journal.pmed.1002210)
Supplement: S2 Text — (DOCX) [file pmed.1002210.s002.docx]

**CONSORT 2010 checklist of information for PACE-UP cluster randomised controlled trial**

| Section/Topic | Item No | Standard Checklist item | Extension for cluster designs | Page No * |
| --- | --- | --- | --- | --- |
| Title and abstract | | | |  |
|  | 1a | Identification as a randomised trial in the title  YES | Identification as a cluster randomised trial in the title  Yes | Title |
|  | 1b | Structured summary of trial design, methods, results, and conclusions (for specific guidance see CONSORT for abstracts)^[[1]](#endnote-1),^^[[2]](#endnote-2)^  YES | See table 2  YES see table 2 below | Abstract |
| Introduction | | | |  |
| Background and objectives | 2a | Scientific background and explanation of rationale  YES | Rationale for using a cluster design  YES | Introduction, paragraphs 1-3 |
|  | 2b | Specific objectives or hypotheses  YES | Whether objectives pertain to the cluster level, the individual participant level or both  YES | Introduction, paragraph 3 |
| Methods | | | |  |
| Trial design | 3a | Description of trial design (such as parallel, factorial) including allocation ratio  YES | Definition of cluster and description of how the design features apply to the clusters  YES | Methods, Study Design and Participants section |
|  | 3b | Important changes to methods after trial commencement (such as eligibility criteria), with reasons  YES |  | Methods, Statistical Analysis section |
| Participants | 4a | Eligibility criteria for participants  YES | Eligibility criteria for clusters  YES | Methods, Study Design and Participants section |
|  | 4b | Settings and locations where the data were collected  YES |  | Methods, Study Design and Participants section |
| Interventions | 5 | The interventions for each group with sufficient details to allow replication, including how and when they were actually administered  YES | Whether interventions pertain to the cluster level, the individual participant level or both  YES | Methods, Procedures section and S1 Fig and published protocol paper (S1 Text) |
| Outcomes | 6a | Completely defined pre-specified primary and secondary outcome measures, including how and when they were assessed  YES | Whether outcome measures pertain to the cluster level, the individual participant level or both  YES | Methods, Outcomes section |
|  | 6b | Any changes to trial outcomes after the trial commenced, with reasons |  | Not applicable |
| Sample size | 7a | How sample size was determined  YES | Method of calculation, number of clusters(s) (and whether equal or unequal cluster sizes are assumed), cluster size, a coefficient of intracluster correlation (ICC or *k*), and an indication of its uncertainty  YES | Methods, Statistical Analysis section and published protocol paper (S1 Text) |
|  | 7b | When applicable, explanation of any interim analyses and stopping guidelines |  | Not applicable |
| Randomisation: | | | |  |
| Sequence generation | 8a | Method used to generate the random allocation sequence  YES |  | Methods, Randomisation and Masking section |
|  | 8b | Type of randomisation; details of any restriction (such as blocking and block size)  YES | Details of stratification or matching if used  YES | Methods, Randomisation and Masking section |
| Allocation concealment mechanism | 9 | Mechanism used to implement the random allocation sequence (such as sequentially numbered containers), describing any steps taken to conceal the sequence until interventions were assigned  YES | Specification that allocation was based on clusters rather than individuals and whether allocation concealment (if any) was at the cluster level, the individual participant level or both  YES | Methods, Randomisation and Masking section |
| Implementation | 10 | Who generated the random allocation sequence, who enrolled participants, and who assigned participants to interventions | Replace by 10a, 10b and 10c |  |
|  | 10a |  | Who generated the random allocation sequence, who enrolled clusters, and who assigned clusters to interventions  YES | Methods, Randomisation and Masking section |
|  | 10b |  | Mechanism by which individual participants were included in clusters for the purposes of the trial (such as complete enumeration, random sampling)  YES | Methods, Randomisation and Masking section |
|  | 10c |  | From whom consent was sought (representatives of the cluster, or individual cluster members, or both), and whether consent was sought before or after randomisation  YES | Methods, Procedures section and published protocol paper (S1 Text) |
|  |  |  |  |  |
| Blinding | 11a | If done, who was blinded after assignment to interventions (for example, participants, care providers, those assessing outcomes) and how  YES |  | Methods, Randomisation and Masking section |
|  | 11b | If relevant, description of the similarity of interventions |  | Methods, Procedures section and S1 Fig and published protocol paper (S1 Text) |
| Statistical methods | 12a | Statistical methods used to compare groups for primary and secondary outcomes  YES | How clustering was taken into account  YES | Methods, Statistical Analysis section |
|  | 12b | Methods for additional analyses, such as subgroup analyses and adjusted analyses  YES |  | Methods, Statistical Analysis section |
| Results | | | |  |
| Participant flow (a diagram is strongly recommended) | 13a | For each group, the numbers of participants who were randomly assigned, received intended treatment, and were analysed for the primary outcome  YES | For each group, the numbers of clusters that were randomly assigned, received intended treatment, and were analysed for the primary outcome  NO | Figure 1 (CONSORT diagram) |
|  | 13b | For each group, losses and exclusions after randomisation, together with reasons  YES | For each group, losses and exclusions for both clusters and individual cluster members  NO | Figure 1 (CONSORT diagram) |
| Recruitment | 14a | Dates defining the periods of recruitment and follow-up YES |  | Methods, Study Design and Participants section |
|  | 14b | Why the trial ended or was stopped |  | Not applicable |
| Baseline data | 15 | A table showing baseline demographic and clinical characteristics for each group  YES | Baseline characteristics for the individual and cluster levels as applicable for each group  YES (for individuals for each group) | Table 1 from paper |
| Numbers analysed | 16 | For each group, number of participants (denominator) included in each analysis and whether the analysis was by original assigned groups  YES | For each group, number of clusters included in each analysis  NO (but not necessary, as individual analyses, taking account of clustering in analyses) | Results, Participant section and CONSORT diagram Figure 1 |
| Outcomes and estimation | 17a | For each primary and secondary outcome, results for each group, and the estimated effect size and its precision (such as 95% confidence interval)  YES | Results at the individual or cluster level as applicable and a coefficient of intracluster correlation (ICC or k) for each primary outcome  YES (results at individual level) | Table 2 from paper |
|  | 17b | For binary outcomes, presentation of both absolute and relative effect sizes is recommended |  | Not a binary outcome |
| Ancillary analyses | 18 | Results of any other analyses performed, including subgroup analyses and adjusted analyses, distinguishing pre-specified from exploratory  YES |  | Results, Table 3, section Sub-group analyses and Figures 2a and 2b and section Sensitivity analyses and imputations and S3 Table |
| Harms | 19 | All important harms or unintended effects in each group (for specific guidance see CONSORT for harms^[[3]](#endnote-3)^)  YES |  | Results section Effect of the intervention on adverse events and serious adverse events and Table 4 |
| Discussion | | | |  |
| Limitations | 20 | Trial limitations, addressing sources of potential bias, imprecision, and, if relevant, multiplicity of analyses  YES |  | Discussion, section Study Strengths and Limitations |
| Generalisability | 21 | Generalisability (external validity, applicability) of the trial findings  YES | Generalisability to clusters and/or individual participants (as relevant)  YES (to individual participants) | Discussion, section Study Strengths and Limitations and section Implications for policy, practice and future research |
| Interpretation | 22 | Interpretation consistent with results, balancing benefits and harms, and considering other relevant evidence  YES |  | Discussion, section Main results in context of other literature |
| Other information | | |  |  |
| Registration | 23 | Registration number and name of trial registry  YES |  | Abstract |
| Protocol | 24 | Where the full trial protocol can be accessed, if available  YES |  | Reference 23 and S1 Text  Open access online journal and available on PACE-UP study website [www.paceup.sgul.ac.uk](http://www.paceup.sgul.ac.uk) |
| Funding | 25 | Sources of funding and other support (such as supply of drugs), role of funders  YES |  | Included in PLoS Med online funding declaration submission |

** Note: page numbers optional depending on journal requirements*

**Table 2: Extension of CONSORT for abstracts**1**^,2^ to reports of cluster randomised trials**

| Item | Standard Checklist item | Extension for cluster trials |
| --- | --- | --- |
| Title | Identification of study as randomised  YES | Identification of study as cluster randomised  Yes |
| Trial design | Description of the trial design (e.g. parallel, cluster, non-inferiority)  YES |  |
| Methods |  |  |
| Participants | Eligibility criteria for participants and the settings where the data were collected  YES | Eligibility criteria for clusters  YES |
| Interventions | Interventions intended for each group  YES |  |
| Objective | Specific objective or hypothesis  YES | Whether objective or hypothesis pertains to the cluster level, the individual participant level or both  YES (individual) |
| Outcome | Clearly defined primary outcome for this report  YES | Whether the primary outcome pertains to the cluster level, the individual participant level or both  YES (individual) |
| Randomization | How participants were allocated to interventions  YES | How clusters were allocated to interventions  YES |
| Blinding (masking) | Whether or not participants, care givers, and those assessing the outcomes were blinded to group assignment  YES |  |
| Results |  |  |
| Numbers randomized | Number of participants randomized to each group  YES | Number of clusters randomized to each group  YES |
| Recruitment | Trial status^^[[4]](#footnote-1)^^ |  |
| Numbers analysed | Number of participants analysed in each group  YES | Number of clusters analysed in each group  NO, not applicable, as analysis at individual level controlling for clustering. |
| Outcome | For the primary outcome, a result for each group and the estimated effect size and its precision  YES | Results at the cluster or individual participant level as applicable for each primary outcome  At individual level |
| Harms | Important adverse events or side effects  NO, this was not a primary or secondary  outcome, but they are reported in paper. |  |
| Conclusions | General interpretation of the results  YES |  |
| Trial registration | Registration number and name of trial register  YES |  |
| Funding | Source of funding  YES |  |
|  |  |  |

**REFERENCES**

1. Hopewell S, Clarke M, Moher D, Wager E, Middleton P, Altman DG, et al. CONSORT for reporting randomised trials in journal and conference abstracts. *Lancet* 2008, 371:281-283 [↑](#endnote-ref-1)
2. Hopewell S, Clarke M, Moher D, Wager E, Middleton P, Altman DG at al (2008) CONSORT for reporting randomized controlled trials in journal and conference abstracts: explanation and elaboration. *PLoS Med* 5(1): e20 [↑](#endnote-ref-2)
3. Ioannidis JP, Evans SJ, Gotzsche PC, O'Neill RT, Altman DG, Schulz K, Moher D. Better reporting of harms in randomized trials: an extension of the CONSORT statement. *Ann Intern Med* 2004; 141(10):781-788. [↑](#endnote-ref-3)
4. Relevant to Conference Abstracts [↑](#footnote-ref-1)
