# Supplementary material for: Effect of a Primary Care Walking Intervention with and without Nurse Support on Physical Activity Levels in 45- to 75-Year-Olds: The Pedometer And Consultation Evaluation (PACE-UP) Cluster Randomised Clinical Trial
Source: PLoS Med. 2017 Jan 3;14(1):e1002210. doi: 10.1371/journal.pmed.1002210 (PMC5207642; doi:10.1371/journal.pmed.1002210)
Supplement: S3 Text — (DOCX) [file pmed.1002210.s003.docx]

**What is the potential benefit of our intervention on Coronary Heart Disease and All-cause mortality?**

Several systematic reviews have assessed the benefits of walking based on pooling data from cohort studies. Typically the relative risks are 0.8 in those who are physically more active compared to those who are much less active. The difficulty of interpreting such analyses is their focus on comparing two extreme groups; the physically active v those inactive. Zheng et al (2009) recognised the importance of studying the functional form of the dose response effect of walking on CHD risk. They concluded that the risk of CHD decreases as amount of brisk walking increases {Zheng et al. Eur J Epidemiol 2009;24:181-92}. Specifically Zheng et al concluded that 150 minutes of brisk walking/wk reduces the incidence of CHD by 19% (a RR of 0.81 (95%CI 0.77, 0.86); relative risk estimates were similar in both sexes and in older and younger subjects. From this we can estimate (see below*) that the increase of 33 minutes per week in the postal group in our study at 12 months would be expected to reduce CHD risk by 4.5% (95%CI 3.3%, 5.6%) if sustained. In a prospective study assessing the benefits of walking in a free living population sample, Dwyer et al 2015 (PLOS ONE 10(11):e0141274) found that higher daily step count measured by pedometer was linearly associated with reductions in all-cause mortality. Using the same method (see ** below) we estimate that the 643 increase in steps in our postal group would result in a 4% (1% to 7%) decrease in mortality.

* From the paper by Zheng et al we can take the fact that log (Risk) increases linearly with minutes of MVPA and that increasing minutes of MVPA/wk by 150 reduces risk by 19% (a relative risk of 0.81), to estimate that increasing MVPA by 33 minutes per week would result in a relative risk of 0.81^(33/150)^ =0 .81^0.22^ (0.77^0.22^, 0.86^0.22^) = 0.955 (0.944, 0.967). That is a 4.5% (3%, 6%) reduction in risk of CHD.

** From the paper by Dwyer et al 2015 (PLOS ONE 10(11):e0141274) the adjusted hazard ratio for all-cause mortality associated with an additional 1000 steps was 0.94 (95% CI .90 to .98). Using the same approach as above an increase of 642 steps is estimated to reduce risk by 0.94^(642/1000)^ = 0.94^(.642)^ (0.90^(.642)^,0.98^(.642)^)= 0.96 (0.93, 0.99). That is a 4% (1% to 7%) reduction in all-cause mortality.

Zheng et al.. Quantifying the dose-response of walkingin reducing coronary heart disease risk: meta-analysis. Eur J Epidemiol 2009;24:181-92

Dwyer et al. Objectively Measured Daily Steps and Subsequent Long Term All-Cause Mortality: The Tasped Perospective Cohort Study. PLoS ONE 2015 10(11):e0141274
