# Supplementary figures and images for: Effect of a Primary Care Walking Intervention with and without Nurse Support on Physical Activity Levels in 45- to 75-Year-Olds: The Pedometer And Consultation Evaluation (PACE-UP) Cluster Randomised Clinical Trial
Source: PLoS Med. 2017 Jan 3;14(1):e1002210. doi: 10.1371/journal.pmed.1002210 (PMC5207642; doi:10.1371/journal.pmed.1002210)

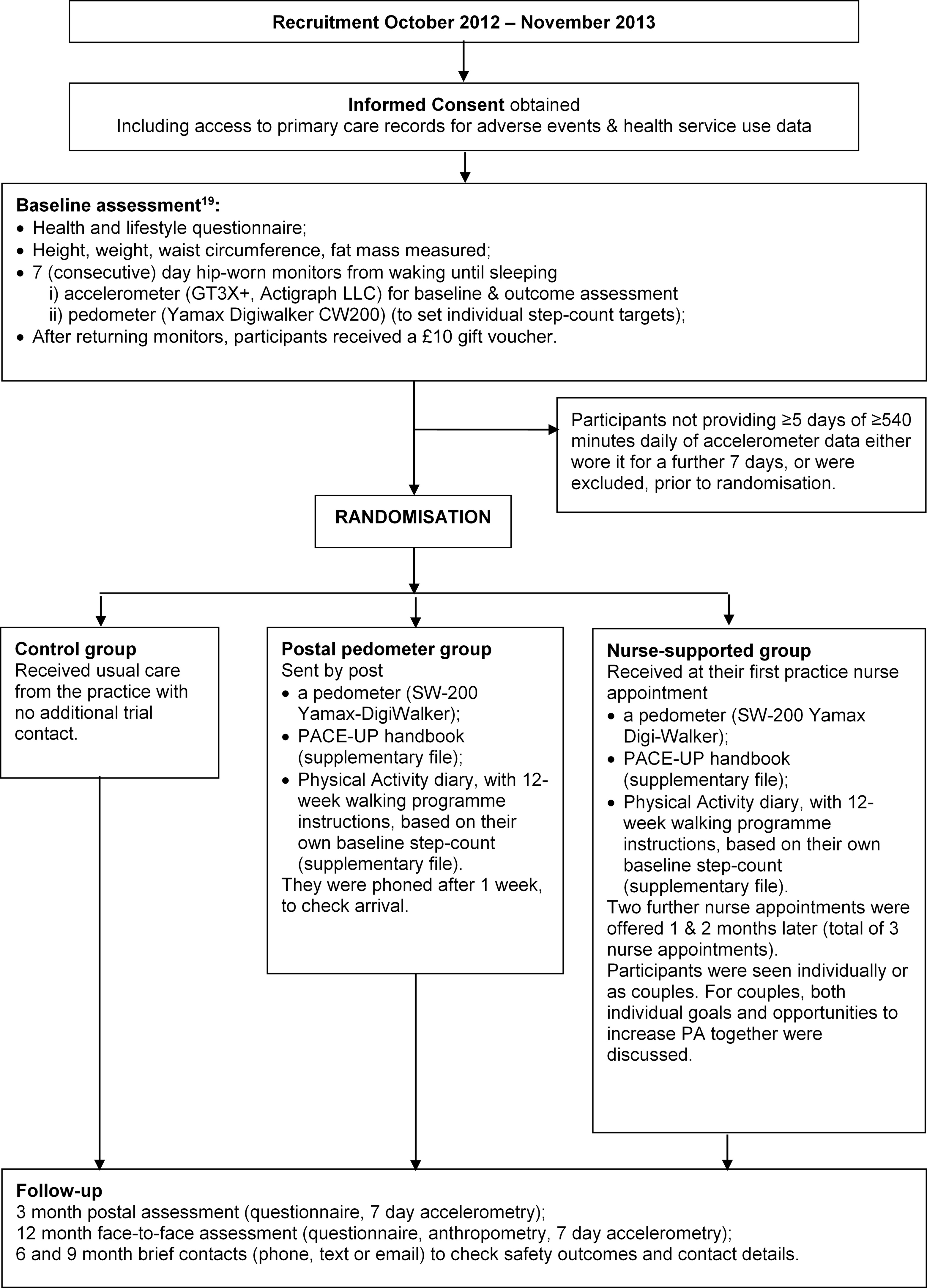

Supplement: S1 Fig — (TIF) [file pmed.1002210.s004.tif]

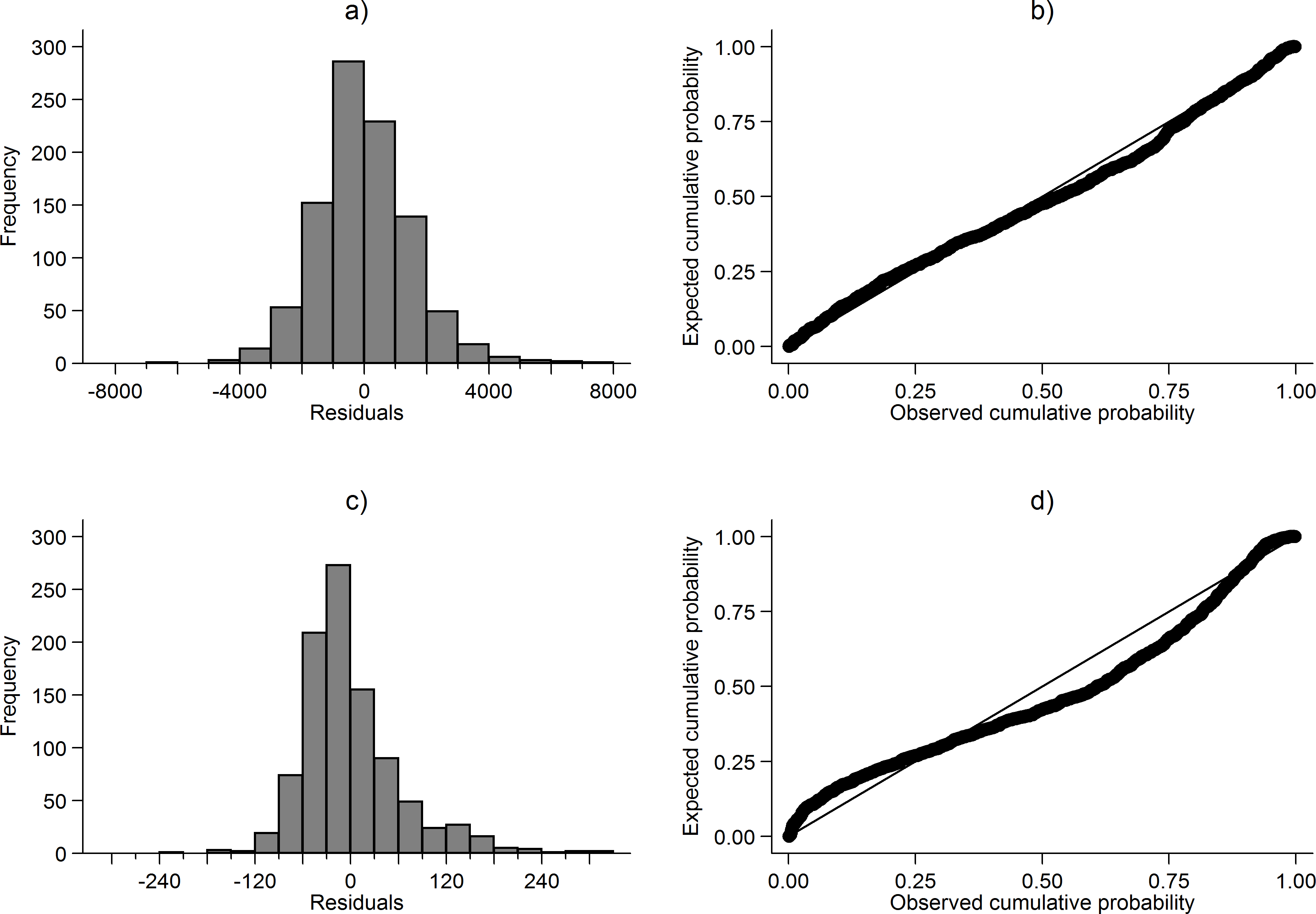

Supplement: S2 Fig — (TIF) [file pmed.1002210.s005.tif]
