# Supplementary material for: Effect of a Primary Care Walking Intervention with and without Nurse Support on Physical Activity Levels in 45- to 75-Year-Olds: The Pedometer And Consultation Evaluation (PACE-UP) Cluster Randomised Clinical Trial
Source: PLoS Med. 2017 Jan 3;14(1):e1002210. doi: 10.1371/journal.pmed.1002210 (PMC5207642; doi:10.1371/journal.pmed.1002210)
Supplement: S1 Table — (DOCX) [file pmed.1002210.s006.docx]

**S1 Table: Number of days with ≥540 minutes accelerometer wear time by treatment group at baseline, 3 months and 12 months**

| **Number of days with ≥540 mins wear time** | **Baseline** | | |  | **3 months** | | |  | **12 months** | | |
| --- | --- | --- | --- | --- | --- | --- | --- | --- | --- | --- | --- |
|  | **Control**  **n=338** | **Postal**  **n=339** | **Nurse**  **n=346** |  | **Control**  **n=318** | **Postal**  **n=317** | **Nurse**  **n=319** |  | **Control**  **n=323** | **Postal**  **n=312** | **Nurse**  **n=321** |
|  |  |  |  |  |  |  |  |  |  |  |  |
| 1 |  |  |  |  | 0 | 2 (0.6%) | 3 (1%) |  | 1 (0.3%) | 0 | 1 (0.3%) |
| 2 |  |  |  |  | 2 (0.6%) | 9 (3%) | 6 (2%) |  | 1 (0.3%) | 1 (0.3%) | 2 (0.6%) |
| 3 |  |  |  |  | 9 (3%) | 8 (3%) | 3 (1%) |  | 5 (2%) | 4 (1%) | 2 (0.6%) |
| 4 |  |  |  |  | 21 (7%) | 16 (5%) | 11 (3%) |  | 16 (5%) | 20 (6%) | 14 (4%) |
| 5 | 29 (9%) | 39 (12%) | 40 (12%) |  | 37 (12%) | 25 (8%) | 35 (11%) |  | 42 (13%) | 38 (12%) | 35 (11%) |
| 6 | 85 (25%) | 83 (24%) | 84 (24%) |  | 64 (20%) | 79 (25%) | 67 (21%) |  | 78 (24%) | 57 (18%) | 79 (25%) |
| 7 | 224 (66%) | 217 (64%) | 222 (64%) |  | 185 (58%) | 178 (56%) | 194 (61%) |  | 180 (56%) | 192 (62%) | 188 (59%) |
| **≥5 days** | **338 (100%)** | **339 (100%)** | **346 (100%)** |  | **286 (90%)** | **282 (89%)** | **296 (93%)** |  | **300 (93%)** | **287 (92%)** | **302 (94%)** |
