# Supplementary material for: Effect of a Primary Care Walking Intervention with and without Nurse Support on Physical Activity Levels in 45- to 75-Year-Olds: The Pedometer And Consultation Evaluation (PACE-UP) Cluster Randomised Clinical Trial
Source: PLoS Med. 2017 Jan 3;14(1):e1002210. doi: 10.1371/journal.pmed.1002210 (PMC5207642; doi:10.1371/journal.pmed.1002210)
Supplement: S2 Table — (DOCX) [file pmed.1002210.s007.docx]

**Table S2. Summary data for main outcome and ancillary outcome variables**

|  | **Control group (mean (sd))** | | | | | |  | **Postal group (mean (sd))** | | | | | |  | **Nurse group (mean (sd))** | | | | | |
| --- | --- | --- | --- | --- | --- | --- | --- | --- | --- | --- | --- | --- | --- | --- | --- | --- | --- | --- | --- | --- |
|  | **Baseline** | | **3 months** | | **12 months** | |  | **Baseline** | | **3 months** | | **12 months** | |  | **Baseline** | | **3 months** | | **12 months** | |
|  |  |  |  |  |  |  |  |  |  |  |  |  |  |  |  |  |  |  |  |  |
| **Accelerometry data (N)** | 338 | | 318 | | 323 | |  | 339 | | 317 | | 312 | |  | 346 | | 319 | | 321 | |
| Daily step count | 7379 | (2696) | 7327 | (2688) | 7246 | (2671) |  | 7402 | (2476) | 8086 | (3014) | 8010 | (2922) |  | 7653 | (2826) | 8707 | (3206) | 8131 | (3228) |
| Total weekly mins of MVPA in ≥10 minute bouts | 84 | (97) | 87 | (101) | 89 | (94) |  | 92 | (90) | 136 | (125) | 129 | (124) |  | 105 | (116) | 164 | (154) | 138 | (141) |
| Daily sedentary time (mins) | 613 | (68) | 614 | (70) | 616 | (72) |  | 614 | (71) | 614 | (74) | 617 | (71) |  | 619 | (78) | 613 | (77) | 620 | (79) |
| Daily wear time (mins) | 789 | (73) | 795 | (78) | 791 | (76) |  | 787 | (78) | 798 | (84) | 800 | (80) |  | 797 | (84) | 805 | (85) | 807 | (89) |
|  |  |  |  |  |  |  |  |  |  |  |  |  |  |  |  |  |  |  |  |  |
| **Clinical measurements (N)** | 338 | |  |  | 323 | |  | 339 | |  |  | 314 | |  | 346 | |  |  | 321 | |
| BMI (kg/m^2^) | 27.7 | (5.4) |  |  | 27.5 | (5.2) |  | 28 | (5.5) |  |  | 27.7 | (5.6) |  | 27.6 | (5.2) |  |  | 27.5 | (5.2) |
| Fat mass (kg) | 26 | (10.3) |  |  | 25.8 | (9.8) |  | 26.8 | (11.0) |  |  | 26.5 | (11.2) |  | 26 | (10.6) |  |  | 25.6 | (11.1) |
| Waist circumference (cm) | 93.1 | (14.3) |  |  | 93.4 | (14.7) |  | 94.1 | (13.9) |  |  | 94.3 | (14.1) |  | 93.2 | (13.2) |  |  | 93.7 | (13.4) |
|  |  |  |  |  |  |  |  |  |  |  |  |  |  |  |  |  |  |  |  |  |
| **Questionnaire data (N)** | 335 | | 316 | | 318 | |  | 335 | | 312 | | 311 | |  | 342 | | 310 | | 319 | |
| HADS Anxiety score | 4.8 | (3.3) | 4.7 | (3.4) | 4.7 | (3.4) |  | 4.6 | (3.3) | 4.4 | (3.3) | 4.4 | (3.4) |  | 4.6 | (3.6) | 4.4 | (3.5) | 4.5 | (3.9) |
| HADS Depression score | 3.9 | (2.8) | 2.7 | (2.6) | 2.6 | (2.9) |  | 3.8 | (2.6) | 2.4 | (2.7) | 2.4 | (2.6) |  | 3.9 | (2.9) | 2.4 | (2.9) | 2.6 | (3.0) |
| EQ5D score | 0.8 | (0.1) | 0.8 | (0.1) | 0.8 | (0.2) |  | 0.9 | (0.1) | 0.9 | (0.1) | 0.8 | (0.1) |  | 0.9 | (0.1) | 0.8 | (0.1) | 0.8 | (0.1) |
| Exercise self-efficacy score | 22.3 | (7.0) | 20.4 | (6.7) | 21.0 | (7.1) |  | 22.1 | (7.2) | 21.4 | (6.7) | 21.7 | (7.0) |  | 22.0 | (7.3) | 22.9 | (6.7) | 22.4 | (7.1) |
| Self-report pain | 0.9 | (0.8) | 0.9 | (0.8) | 1.0 | (0.8) |  | 1.0 | (0.8) | 1.0 | (0.8) | 1.0 | (0.8) |  | 0.9 | (0.8) | 1.0 | (0.8) | 1.0 | (0.8) |
|  |  |  |  |  |  |  |  |  |  |  |  |  |  |  |  |  |  |  |  |  |

**Footnotes**Accelerometry data are adjusted for day of the week and day order of wearing the accelerometer with participant as a random effect in a multi-level model.

At baseline, data were available for all participants for accelerometry variables, BMI and waist circumference. Fat mass was available for 335, 337 and 346 participants in the control, postal and nurse groups respectively. There were no clinical measurements at 3 months. At 12 months, fat mass was available in the control, postal and nurse groups respectively for 319, 308 and 320 participants.

Questionnaire variables were missing for varying numbers of participants at each time-point.

Full references for HADS anxiety and depression score, EQ5D score and exercise self-efficacy are given in the trial protocol ^23^
