# Supplementary material for: Effect of a Primary Care Walking Intervention with and without Nurse Support on Physical Activity Levels in 45- to 75-Year-Olds: The Pedometer And Consultation Evaluation (PACE-UP) Cluster Randomised Clinical Trial
Source: PLoS Med. 2017 Jan 3;14(1):e1002210. doi: 10.1371/journal.pmed.1002210 (PMC5207642; doi:10.1371/journal.pmed.1002210)
Supplement: S3 Table — (DOCX) [file pmed.1002210.s008.docx]

**S3 Table: Sensitivity and imputation analyses for the primary outcome (step count at 12 months)**

|  |  | **Postal vs control** | | | **Nurse vs Control** | | | **Nurse vs Postal** | | |
| --- | --- | --- | --- | --- | --- | --- | --- | --- | --- | --- |
|  | **N** | **Effect** | **95% CI** | ***p*-value** | **Effect** | **95% CI** | ***p*-value** | **Effect** | **95% CI** | ***p*-value** |
| **Analysis based on all participants with follow-up data.** | |  |  |  |  |  |  |  |  |  |
| Minimum daily wear time 540 minutes |  |  |  |  |  |  |  |  |  |  |
| At least 5 days at baseline and 1 day at 12 months | 956 | 642 | (329, 955) | <0.001 | 677 | (365, 989) | <0.001 | 36 | (-277, 349) | 0.82 |
| At least 5 days at baseline and 5 days at 12 months | 889 | 607 | (285, 930) | <0.001 | 732 | (412, 1051) | <0.001 | 124 | (-198, 446) | 0.45 |
|  |  |  |  |  |  |  |  |  |  |  |
| Minimum daily wear time 600 minutes |  |  |  |  |  |  |  |  |  |  |
| At least 5 days at baseline and 1 day at 12 months | 877 | 675 | (352, 997) | <0.001 | 714 | (392,1036) | <0.001 | 39 | (-283, 362) | 0.81 |
| At least 5 days at baseline and 5 days at 12 months | 760 | 752 | (411, 1093) | <0.001 | 796 | (456, 1136) | <0.001 | 44 | (-295, 384) | 0.80 |
|  |  |  |  |  |  |  |  |  |  |  |
| Model adjusting for change in wear time between baseline and 12 months | 956 | 579 | (273, 885) | <0.001 | 637 | (332, 941) | <0.001 | 58 | (-248, 363) | 0.71 |
|  |  |  |  |  |  |  |  |  |  |  |
| **Analyses based on all randomised participants: missing step counts imputed for participants with no follow-up data at 12 months** | | | | | | | |  |  |  |
| 1. Missing at random | |  |  |  |  |  |  |  |  |  |
| Imputed using treatment group, baseline steps, gender, age, practice, month baseline accelerometry | 1023 | 638 | (324, 953) | <0.001 | 679 | (367, 992) | <0.001 | 41 | (-270, 352) | 0.80 |
|  |  |  |  |  |  |  |  |  |  |  |
| Imputed using treatment group, baseline steps, gender, age, practice, month baseline accelerometry, NS-SEC, self-reported pain and fat mass^†^ | 1013 | 673 | (356, 989) | <0.001 | 686 | (372, 1000) | <0.001 | 13 | (-303, 330) | 0.94 |
|  |  |  |  |  |  |  |  |  |  |  |
| 2. Missing not at random using extreme assumptions for missing data: | | |  |  |  |  |  |  |  |  |
| Control group: 12 month step count equal to baseline step count  Both intervention groups: 12 month step count changes by: | | | | |  |  |  |  |  |  |
| -1500 steps | 1023 | 651 | (338, 964) | <0.001 | 783 | (472, 1095) | <0.001 | 132 | (-181, 445) | 0.41 |
| Same as baseline step count | 1023 | 771 | (458, 1084) | <0.001 | 892 | (580, 1204) | <0.001 | 121 | (-192, 434) | 0.45 |
| +1500 steps | 1023 | 890 | (577, 1203) | <0.001 | 1000 | (688, 1312) | <0.001 | 110 | (-203, 423) | 0.49 |
|  |  |  |  |  |  |  |  |  |  |  |

**Footnotes**

† Of the 67 participants with inadequate accelerometry at 12 months, baseline data for NS-SEC were also missing for 10 participants and so imputed values were not available for these 10 participants when including NS-SEC as a predictor.
